# Physical activity and mental health in women with Polycystic Ovary Syndrome

**DOI:** 10.1186/1472-6874-14-51

**Published:** 2014-03-27

**Authors:** Lauren K Banting, Melanie Gibson-Helm, Remco Polman, Helena J Teede, Nigel K Stepto

**Affiliations:** 1 Institute of Sport Exercise and Active Living, Victoria University, Melbourne, Victoria, Australia; 2 Women’s Public Health Research, Monash Centre for Health Research and Implenetation, School of Public Health and Preventive Medicine, Monash University, Clayton, Victoria, Australia; 3 College of Sport and Exercise Science, Victoria University, PO Box 14428, Melbourne, Victoria 8001, Australia; 4 Diabetes and Vascular Medicine Unit, Monash Health, Clayton, Melbourne, Victoria, Australia

**Keywords:** Depression, Anxiety, Exercise, Motivation, Chronic disease, PCOS, Polycystic ovary syndrome

## Abstract

**Background:**

Physical activity is prescribed as a component of primary management for Polycystic Ovary Syndrome (PCOS). This study investigates the association between physical activity and mental health as well as the exercise barriers, motivators and support providers for younger women with and without PCOS to assist in physical activity uptake and prescription for these women.

**Methods:**

Women aged 18-50 years with (n = 153) and without PCOS (n = 64) completed a questionnaire at one time point. The questionnaire included the Hospital Anxiety and Depression Scale and a survey regarding levels of physical activity, physical activity barriers, motivators and supports. A MANCOVA assessed associations between physical activity, PCOS and mental health (specifically depression and anxiety). Descriptive and Chi square goodness of fit statistics assessed the differences in perceived barriers, motivators and support providers amongst women with and without PCOS.

**Results:**

Women with PCOS displayed higher severity of depression (*F(1,210*) = 8.32, *p* = 0.004) and anxiety (*F(1,210)* = 17.37, *p* < 0.001) symptoms compared to controls. Overall, for physically active women, depression was significantly less severe than in their inactive counterparts (*F(2,210)* = 13.62, *p <* 0.001). There were no differences in anxiety by physical activity status and no interaction effects between PCOS and activity status for depression or anxiety. Women with PCOS were more likely to report a lack of confidence about maintaining physical activity *(Χ*^*2*^ = 3.65; *p =* 0.046), fear of injury *(Χ*^*2*^ = 4.08; *p =* 0.043) and physical limitations *(Χ*^*2*^ = 11.92; *p =* 0.001) as barriers to physical activity and were more likely to be motivated to be active to control a medical condition *(Χ*^*2*^ = 7.48; *p =* 0.006). Women with PCOS identified more sources of support compared to women without PCOS.

**Conclusions:**

Physical activity is associated with lower depression in women with PCOS and differences exist in the self-reported physical activity barriers, motivators and support providers, compared to controls. Being more active may offer mental health benefits in managing PCOS. Prescribing physical activity to women with PCOS should be individualized and consider both common and PCOS-specific barriers and motivators for successful engagement.

## Background

Polycystic ovary syndrome (PCOS) is one of the most common and complex endocrine disorders and the leading cause of anovulatory infertility in reproductive aged women. PCOS affects between 12-21% of reproductive aged women, depending on diagnostic criteria, with many cases being undiagnosed [1,2]. PCOS has reproductive, psychological and cardio-metabolic features [3-7] and is associated with many long term adverse health problems including increased risk of obesity [4,8,9], type 2 diabetes and metabolic impairments [10-13], and cardiovascular risk factors [14,15]. We and others have shown that diminished mental health is related to PCOS, including depression, anxiety and lower quality of life [16-18]. These mental health differences are noted across the lifespan, including adolescents, and across the different PCOS phenotypes [19,20].

Many chronic illnesses have mental health impacts and are associated with reduction in quality of life and increases in depressive symptoms [21-24]. In PCOS, symptoms and co-morbidities increase the risk of adverse mental health consequences. Coping with the condition itself, fears regarding infertility, loss of femininity and sexuality, body image concerns and lower self-worth may all contribute to poorer mental health outcomes [7,18,25]. Mental health is especially relevant in PCOS management as it is vital to self-efficacy around a healthy lifestyle (including physical activity). Therefore optimisation of physical activity as a treatment for PCOS, as recommended by the first Evidence based Guideline for the Assessment and Management of PCOS [26], should consider the mental health status of women with PCOS and the interactions with physical activity.

Lifestyle management, including healthy diet and physical activity, is currently advised as the first line management strategy for PCOS [26]. Physical activity is an effective therapeutic option for the reproductive [27,28] and metabolic features [29] of PCOS. The specific interaction between physical activity and mental health has not been explored in depth in PCOS, however, preliminary data including a recent clinic-based study of women with PCOS found physically inactive women had higher depression scores than physically active women, and there was an association between lower physical activity and mild depression [30]. Reductions in depression and elevations in quality of life have been observed in combined physical activity and dietary interventions in PCOS [31] and specific physical activity interventions have been found to be effective in reducing mild-moderate depression [32,33] and anxiety [34] in the general population. Yet engagement and sustainability in physical activity in women with PCOS is particularly problematic. Despite the positive physical effects of exercise [35], the inherent symptoms and co-morbidities associated with PCOS may present barriers to engagement in physical activity and may impact on typically positive psychological responses to being physically active. Thus, research investigating the relationship between physical activity and mental health for women with PCOS and exploration of the enablers and the barriers to activity is important for appropriate prescription of physical activity Additionally, as attrition in PCOS physical activity research interventions is typically high (30-40%) [29,31,35] and physical activity maintenance in PCOS is generally inadequate, understanding the barriers and motivators of physical activity for women with PCOS is required to promote engagement and sustainability. Little is known about the barriers and motivators of physical activity that specifically effect women with PCOS, and being the first study to explore physical activity barriers and motivators among this population of women, the study was considered hypothesis generating.

We hypothesised that physical activity would be associated with better mental health, specifically a lower severity of both anxiety and depression, for all women. Furthermore, the study aimed to describe the common barriers and motivators to physical activity for women with PCOS compared to a control sample of women. We expected some barriers and motivators to differ between women with and without PCOS and that they may vary according to age [36].

## Methods

### Participants

This sub-study focused on physical activity as part of a broader study investigating health-related behaviour in women with and without PCOS. Study recruitment has been described in detail previously [18]. This was a cross-sectional, observational study utilising devised and validated questionnaires. Southern Health Human Research Ethics Committee C (Project No. 07070C) approved this study and the procedures involved. All women provided informed consent to participate in the study. Women responded to recruitment advertisements across a range of community health settings including medical clinics, print media, online women’s health sites and an online PCOS support group. Women needed to be living in Australia, aged between 18 and 70 years and able to read and write in English. Women with and without PCOS were recruited in this manner. PCOS status was then determined based on self-reported previous medical diagnosis (Rotterdam criteria) [37] and the presence of at least two of the following (self-reported during phone screening); polycystic ovaries on ultrasound, high androgen levels/clinical hyperandrogenism and menstrual irregularity. Only the women aged 18- 50 years were included for analysis to reduce any potential confounding effects of menopause (average age of onset is 51 years) which is associated with mental health outcomes [38]. Pregnant women, women living overseas, women with a psychiatric illness (other than anxiety or depression) or an incomplete PCOS diagnosis were excluded from the research. Participants completed the survey in either online or written format according to their preference.

### Measures

#### Body mass index

BMI was calculated based on self-reported weight and height (weight/height(m)^2^). This population (adult women) is known to typically underestimate their weight and overestimate their height (therefore reducing estimated BMI) [39], however, any adjustments required would be common to both groups.

#### Physical activity

A simple, devised measure based on the trans-theoretical model TTM; [40] was used to assess participants’ physical activity levels. As this was part of a large study exploring many aspects of health related behaviour this tool was used rather than longer physical activity tools or pedometers to reduce the burden on participants while gaining a general indication of the individual’s habitual physical activity. A definition of physical activity based on the National Physical Activity Guidelines for Australians was provided to participants. These guidelines define physical activity in a very similar way to other organisations including the World Health Organisation [41]. Participants selected a statement of agreement with each phrase typically used to describe the pre-contemplation (*I currently do not exercise and I don’t intend to start exercising any time soon)*, contemplation (*I currently do not do exercise however I am thinking about exercising in the next few months)*, preparation (*I occasionally exercise now, but not on a regular basis*), action (*I currently exercise on a regular basis and have done so for the past 6 months)* and maintenance (*I currently exercise on a regular basis and have done so for more than 6 months)* stages of the TTM. Responses ranged from 1 (*Definitely agr*ee) to 4 (*Definitely disagree*). Women were classified as physically active if they reported agreement (definite or somewhat agreement) for the active or maintenance stages of the TTM questionnaire.

#### Mental health measures

The Hospital Anxiety and Depression Scale HADS; [42] is a state measure of anxiety and depression, used to detect clinical cases and the severity of both conditions. The scale consists of 14 items, 7 measuring depression, 7 measuring anxiety. The measure displays high internal consistency for both anxiety (α = .93) and depression (α = .90) [43]. The scores can be used as a continuous variable, with higher scores (out of a possible 21) indicating greater anxiety or depression. A score of 8 or greater indicates possible presence of anxiety and/or depression.

#### Physical activity barriers and motivators questionnaire

Women were presented with two lists comprised of commonly reported physical activity barriers and motivators. Participants were asked to nominate which of the barriers were ‘main factors that prevent/stop’ them from engaging in physical activity. The items included 12 barriers commonly associated with physical activity. Participants could also nominate barriers in the ‘other’ category. This option resulted in ‘no enjoyment’ being analysed as a 13^th^ barrier. For the motivators, participants were asked to identify the items which they considered to be ‘the main advantages of engaging in physical activity’. Items included 13 motivators frequently cited as reasons for physical activity and again, participants could nominate other items which resulted in ‘enjoyment/fun’ and ‘time to think’ being included for analysis. The barriers and motivators were selected from international physical activity literature with specific local barriers and motivators selected from the Main Barriers and Motivators Report by the Australian Bureau of Statistics [44].

#### Physical activity supports

From a list of 9, women were asked to select individuals or groups that ‘are most important to you when you think about engaging in physical activity’. These supports were chosen from previous literature and current Australian physical activity reports concerning physical activity facilitation [44]. Women could select as many as relevant and nominate in an ‘other’ category, resulting in ‘myself’ , ‘no one’ , ‘exercise acquaintances or team mates’ and ‘pets’ being included for analysis.

### Data analysis

#### Statistical analysis

Analysis was performed using SPSS version 19. The outcome variables, anxiety and depression, were determined to be normally distributed. A two-way between groups MANCOVA was conducted to determine significant mean differences in mental health (anxiety and depression) amongst the sample according to physical activity (active vs. inactive) and PCOS (PCOS vs. non PCOS). Age and BMI were used as covariates given the significant role each variable has been shown to have on mental health [38,45]. Bonferroni adjustments were used to assess statistical significance for single dependent variables (two-sided *p*-value of 0.025). Independent *t*-tests were used to assess individual differences according to PCOS and physical activity. Numbers of physically active and inactive women with and without PCOS were assessed using a chi-square goodness of fit analysis with statistical significance assumed with a two-sided *p* value of 0.05. Barriers, motivators and physical activity supports were assessed using descriptive statistics, calculating percentages of each group reporting the barrier as a main factor that prevents physical activity which were compared using chi-square goodness of fit analyses. Results were assessed across the entire sample and also considering age groups (18-30 years, 31-40 years and 41-50 years to represent typical life stages) to determine whether barriers, motivators and supports differed. The barriers, motivators and supports were also compared between groups in only healthy weight women (BMI = 18-25). The number of barriers, motivators and supports identified by each group was compared using an independent samples *t*-test. Statistical significance was assumed with a two-sided *p*-value of 0.05.

## Results

### Participation and demographics

After considering the inclusion criteria, 240 women were included for analysis. Twenty three (9.6%) women were excluded from further analysis due to inconsistent responses to the TTM questions for physical activity. Women who indicated agreement with both inactive and active stages (or disagreement with all stages) were removed from analysis as this response pattern indicates a misunderstanding of the question, or erratic physical activity behaviour. The remaining sample (*N* = 217) were included in the analysis and had a mean age of 31.84 years (*SD* = 7.74) and were on average overweight with a mean body mass index (BMI) of 29.23 (*SD* = 7.90). Women with PCOS accounted for 70.5% of the sample (*n* = 153) and had a higher mean BMI than the control group (Table 1). Of the 40 women born overseas 17 (43%) were born in Europe (United Kingdom of Great Britain, Italy, Portugal, Netherlands), 11 (28%) were born in Asia (Hong Kong, India, Sri Lanka, Indonesia, Laos, Malaysia, Singapore), 5 (12%) were born in Africa (Kenya, Zimbabwe, South Africa), 5 (12%) were born in Oceania (New Zealand, Papua New Guinea) and 2 (5%) were born in America (Chile, United States of America).

**Table 1:** Descriptive statistics according to PCOS status

	**PCOS n = 153**	**Control n = 64**	***P-*** **value**
**Age (SD)**	31.99 (6.80)	31.50 (9.69)	0.674
**BMI (kg/m**^**2**^**)**	31.32 (7.71)	24.15 (5.77)	<0.001
**Diagnosed infertility (%)**	37	0	-
**Highest level of education**			0.897
**No formal qualification (%)**	13	17	
**Formal qualification**	87	83	
**Born in Australia (%)**	88	67	<0.001
**Children (% with child)**	43	27	0.027
**Living in urban setting (%)**	75	80	0.444
**Living arrangements**			0.034
**Living alone (%)**	5	13	
**With partner only (%)**	31	30	
**With partner and children (%)**	37	19	
**With children only (%)**	3	2	
**Other adults (%)**	24	38	
**Income**			0.178
**Below $59,999 (%)**	26	24	
**$60,000- $79,999 (%)**	21	17	
**$80,000 + (%)**	46	48	
**Don’t know/no answer (%)**	7	11	

### Physical activity, anxiety and depression

**Figure 1:**
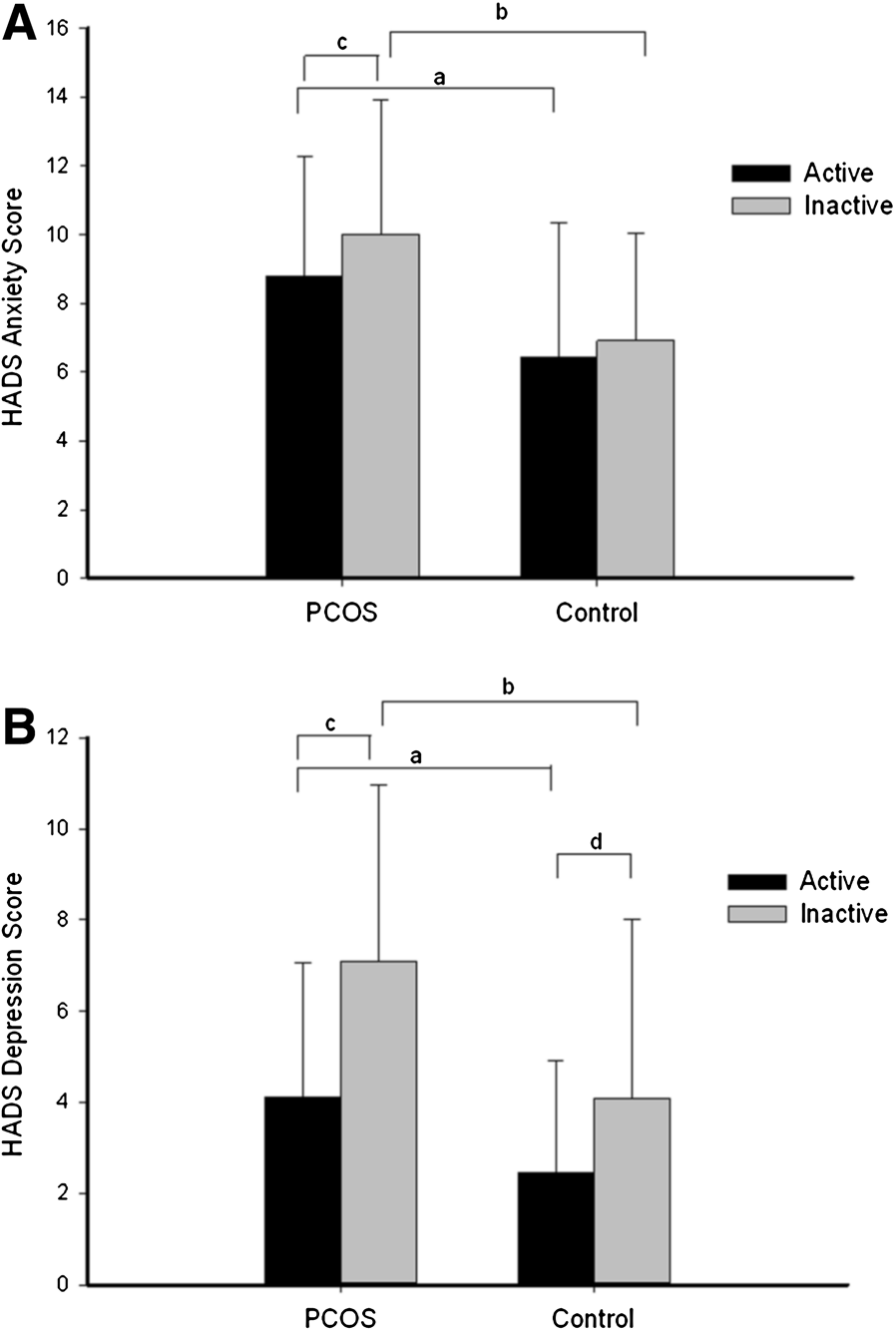
**Mental health according to PCOS status and activity. Panel A**: Mean HADS Anxiety Score according to PCOS status and activity.** Panel B**: Mean HADS Depression Score according to PCOS status and activity. Error bars represent standard deviations, ^a^significant difference in PCOS compared to control for active women (*p*<0.01); ^b^significant difference in PCOS compared to control for inactive women (*p*<0.01); ^c^significant difference in active compared to inactive for PCOS women (*p*<0.05); ^d^significant difference in active compared to inactive for control women (*p*= 0.05).

The Chi-square goodness of fit analysis indicated that more women without PCOS (64%) were physically active than women with PCOS (48%) (*p* = 0.028). After covarying/accounting for age and BMI, both physical activity level (Wilk’s Lambda = 0.93) and PCOS status (Wilk’s Lambda = 0.92) were significantly associated with the mental health of the participants (combined dependent variable of anxiety and depression). BMI had a significant independent influence on mental health with higher BMI associated with poorer mental health (Wilks’s Lambda =0 .94) yet no effect was observed for the covariate of age. Women with PCOS (mean = 9.42 [SD = 3.76]) had higher anxiety scores than women without PCOS (6.60 [3.63]). This difference was significant (*F(1,210)* = 17.37, *p* < 0.001, partial η^2^ = 0 .08). Women with PCOS (5.67 [3.76]) also had higher depression scores than women without PCOS (3.05 [3.15]), a difference which was also significant (*F(1,210)* = 8.32, *p* = 0 .004, partial η^2^ = 0.04). Activity level was not associated with anxiety (*F(2,210)* = 1.79, *p* = 0.18, partial η^2^ = 0.01) but was significantly associated with depression (*F(2,210)* = 13.62, *p* < 0.001, partial η^2^ = 0.06). Physically active women (3.53 [2.88]) had significantly lower depression scores than inactive women (6.41 [4.07]). No significant effects were observed for the interaction of PCOS status and activity level. The descriptive statistics according to group and physical activity are presented in Figure 1.

### Exercise barriers

Table 2 details the exercise barriers identified by women with and without PCOS. The number of exercise barriers amongst women with and without PCOS was similar (*t* = 1.54, *p =* 0.216) with each group identifying approximately four barriers. The barriers were assessed according to age groups (18-30, 31- 40 and 41-50 years) and were determined to be similar in terms of the five most frequently reported exercise barriers and so results are presented for the whole sample. Lack of time and fatigue were the main exercise barriers for both groups. Women with PCOS were more likely to report a lack of confidence regarding their ability to maintain exercise as a barrier (71% of PCOS women, 50% of control women, *p* = 0.046). This barrier was the third most commonly cited barrier for both groups. Women with PCOS were also more likely to report fear of injury and physical limitations as barriers to exercise compared to the women in the control group.

**Table 2:** Barriers to exercise for women with and without PCOS

**Barrier**	**PCOS (%)**	**Control (%)**	***Χ***^***2 ***^ ***(p)***
Lack of time	121 (79)	46 (72)	0.33 (0.569)
Fatigue	118 (77)	44 (69)	0.44 (0.508)
Not confident can maintain*	109 (72)	32 (50)	3.97 (0.046)
Tiredness	57 (37)	16 (25)	2.32 (0.128)
Fear of injury*	47 (31)	11 (17)	4.08 (.043)
Physical limitations*	45 (29)	5 (8)	11.92 (0.001)
Bad weather	45 (29)	20 (31)	0.07 (0.796)
No motivation	40 (26)	24 (38)	2.25 (0.134)
Not important	39 (25)	13 (20)	0.56 (0.456)
Cost	26 (17)	10 (16)	0.03 (0.862)
Young children	17 (11)	4 (6)	1.47 (0.225)
Illness	13 (9)	3 (5)	-
Spousal influence	4 (3)	1 (2)	-
Other	3 (2)	0 (0)	-
No enjoyment	0 (0)	0 (0)	-

*Note:* *indicates significant difference between groups based on Chi-Square goodness of fit analyses (*p*<0.05).

### Exercise motivators

The motivators identified by women with and without PCOS are presented in Table 3. There were no differences between women in terms of PCOS status for the number of motivators reported. Women with PCOS reported no more motivators (*x* = 8.51, *SD* = 3.27) than women without PCOS (*x* = 8.27, *SD* = 2.68)(*t* = 0 .27, *p* = 0.607). Results are presented for the whole sample after the top five motivators were assessed across age groups and were determined to be similar. Weight control, health improvements, increased energy, stress reduction and health maintenance were the most commonly cited motivators in both groups. Controlling a medical condition (PCOS = 40%, Control = 19%, *p* = 0.006) was more commonly cited as an exercise motivator by women with PCOS.

**Table 3:** Exercise motivators for women with and without PCOS

**Motivator**	**PCOS (%)**	**Control (%)**	***Χ***^***2 ***^ ***(p)***
Control weight	144 (94)	52 (81)	0.97 (0.326)
Improve health	142 (93)	49 (77)	1.51 (0.220)
Increase energy	128 (84)	47 (73)	0.77 (0.380)
Stress reduction	123 (80)	47 (73)	0.32 (0.571)
Maintain health	121 (79)	45 (70)	0.54 (0.461)
Improve mood	117 (76)	43 (67)	0.57 (0.452)
Maintain fitness	115 (75)	51 (80)	0.16 (0.688)
Improve self confidence	103 (67)	34 (53)	1.63 (0.201)
Decrease risk of disease	100 (65)	37 (58)	0.40 (0.528)
Decrease risk of illness	71 (46)	31 (48)	0.04 (0.837)
Control medical condition*	61 (40)	12 (19)	7.48 (0.006)
Control blood glucose levels	59 (39)	16 (25)	3.06 (0.080)
Social interaction	54 (35)	16 (25)	1.67 (0.197)
Other	1 (1)	1 (2)	-
Enjoyment	1 (1)	1 (2)	-
Own space	0 (0)	1 (2)	-

*Note:* *indicates significant difference between groups based on Chi-Square goodness of fit analyses (*p*<0.05).

### Physical activity supports

Table 4 summarises the exercise support providers identified by women with and without PCOS. Women with PCOS (x = 3.80, SD = 1.96) were able to identify approximately 1 more support source than women without PCOS (x = 3.20, SD = 1.69) (*t =* 4.19, *p =* 0.041). The 18-30 years old age group reported different support groups to the rest of the sample and so results are presented for this group separately (Table 4). In all groups the husband or partner was the primary support provider, followed by friends and parents. The 18-30 years old age group reported physical activity instructors to be one of the top five support providers, whilst children were primary support providers for the other age groups. For the whole sample, other family were more frequently cited by women with PCOS as support providers (PCOS = 50%, Control = 30%, *p =* 0.025), as were children (PCOS = 43%, Control = 25%, *p* = 0.038).

**Table 4:** Support providers for women with and without PCOS: whole sample

	**Whole sample**			**18- 30 years old sample**		
**Support**	**PCOS (%)**	**Control (%)**	***Χ***^***2 ***^ ***(p)***	**PCOS (%)**	**Control (%)**	***Χ***^***2 ***^ ***(pcp***
Husband/partner^*a*^	116 (76)	37 (58)	2.42 (0.120)	52 (74)	14 (42)	8.83 (0.003)
Friends	93 (61)	30 (47)	1.82 (0.178)	46 (66)	22 (67)	0.01 (0.931)
Parents	86 (56)	24 (38)	3.45 (0.063)	49 (70)	21 (64)	0.27 (0.604)
Other family*	77 (50)	19 (30)	5.00 (0.025)	42 (60)	14 (42)	3.18 (0.075)
Children*^a^	65 (42)	16 (25)	4.31 (0.038)	15 (21)	2 (6)	8.33 (0.004)
Health care profs	42 (27)	14 (22)	0.51 (0.475)	22 (31)	8 (24)	0.89 (0.345)
Co-workers	40 (26)	13 (20)	0.78 (0.376)	24 (34)	9 (27)	0.80 (0.370)
Physical activity instructors	40 (26)	14 (22)	0.33 (0.564)	25 (36)	10 (30)	0.55 (0.460)
Myself	9 (6)	4 (6)	0.00 (1.00)	5 (7)	4 (12)	1.32 (0.251)
Other	4 (3)	0 (0)	-	2 (3)	0 (0)	-
Exercise friends/team	2 (1)	2 (3)	-	2 (3)	0 (0)	-
No one	1 (1)	1 (2)	-	0 (0)	1 (3)	-
Pets	1 (1)	1 (2)	-	1 (1)	0 (0)	-

*Note:* *indicates significant difference between groups in whole sample based on Chi-Square goodness of fit analyses (*p*<0.05).

^*a*^indicates significant difference between groups in 18-30 year old sample based on Chi-Square goodness of fit analyses (*p*<0.05).

## Discussion

In this novel formative research, fewer women with PCOS reported being physically active compared to women without PCOS. Additionally, women with PCOS had poorer mental health, with higher depression and anxiety scores. Relationships between activity and mental health showed that being physically active was associated with lower depression scores, after accounting for age, BMI and PCOS status. There was no interaction effect of PCOS status and physical activity; depression scores were lower in both physically active groups similarly. Physical activity was not significantly associated with lower anxiety after controlling for age, BMI and PCOS status. Women with PCOS did not identify additional barriers or motivators for physical activity, although they did identify an additional supportive influence than women without PCOS. The barriers, motivators and support providers were similar between groups, yet women with PCOS more often reported a lack of confidence in their ability to maintain exercise, fear of injury and physical limitations as barriers to physical activity. These women were also more likely to report being motivated to do physical activity in order to control a medical condition.

As research and practice regarding physical activity prescription as a management strategy for women with PCOS has been significantly limited by poor engagement and sustainability, formative research is important to assess the association between mental health and activity research to explore the physical activity barriers, motivators and support providers for women with PCOS. The current novel findings from a multiethnic community based sample, show that physical activity was associated with lower depression symptoms compared to physical inactivity for all women after accounting for age, BMI and PCOS. Unsurprisingly, both BMI and PCOS were significantly associated with depression as has been found in previous research [19,46]. The current results support findings from a clinic-based study that being physically active is associated with lower rates of depression in women with PCOS [30]. Current results also strengthen claims that participation in physical activity programs may be associated with lower depressive symptoms and improvements in quality of life [31,47] and indicate the value of exploring these associations further using interventional and longitudinal research.

Physical activity was not significantly associated with lower anxiety compared to being inactive after accounting for age, BMI and PCOS. A higher BMI was associated with greater anxiety which is consistent with previous research [46]. Having PCOS was also associated with higher anxiety, supporting the claim that PCOS is associated with poor mental health outcomes [19]. These results are consistent with findings in other populations [48]. Potentially depression may be more associated with and a greater barrier to physical activity than anxiety, with further research needed.

In this sample, physic activity levels in women with PCOS were lower compared to active women without PCOS. Investigation into the barriers and motivators of physical activity in this study emphasised that despite having a medical condition and noting this as a motivator for physical activity, women with PCOS still noted time and fatigue as the key barriers to engaging in physical activity, similar to the control group. Having a positive attitude towards physical activity is insufficient for long-term physical activity engagement, as positive attitudes towards physical activity do not correspond with actual increases in physically active behaviour [49]. Additional factors such as addressing barriers and enhancing support are likely to be needed to increase engagement and sustainability in physical activity in PCOS. In this context, women with PCOS did note some specific barriers which should be considered in physical activity prescription. These included a lack of confidence in their ability to maintain physical activity, fear of injury and physical limitations. Given the role of physical activity in the management of PCOS, a lack of confidence in maintaining physical activity may indicate that women with PCOS need additional support and assistance in sustainable and effective physical activity programs. Physical limitations and fear of injury can be very inhibiting for an individual. Programs and advice may need to consider this barrier and develop strategies to give women with PCOS more confidence in their ability to perform physical activity safely and according to their physical abilities.

Women with and without PCOS noted similar motivators for physical activity. Women with PCOS were more likely to cite controlling a medical condition as a reason for doing physical activity compared to the control group, but only 40% of women with PCOS saw this as a motivator. No significant differences were observed for decreasing risk of disease or illness or controlling blood glucose levels as physical activity motivators between women with or without PCOS. This is despite the fact that women with PCOS have a higher mean BMI and greater risk of diabetes and cardio-metabolic risk factors [50]. This indicates that the importance of physical activity for managing current PCOS symptoms and minimising long-term complications of PCOS may not be fully understood by women with PCOS. As diet and physical activity are endorsed as the primary management strategies for PCOS in recent evidence based guidelines [26] better promotion of short and long term benefits of lifestyle modification is warranted to improve motivation for physical activity.

Women with PCOS indicated more support providers for physical activity than the control group which is positively associated with adherence to lifestyle modifications [49]. Whilst differences were observed across age groups in terms of children being support providers, this is likely to be due to the age of the women themselves and their children in the 18-30 years old group. Support from physical activity instructors and other health professionals could be a way to engage more women with physical activity for a longer time period [49]. This was especially pertinent for women with PCOS aged 18-30 years, among whom one-third identified physical activity instructors and health professionals as important support providers or their physical activity pursuits.

Interestingly, when assessing the barriers, motivators and support providers for physical activity for women with and without PCOS with a healthy BMI, there were no differences between groups (data not shown). Whilst this does indicate that some of the barriers, motivators and support providers may be associated with the higher BMI, this difference may be relevant as women with PCOS are more likely to be overweight, and physical activity prescription should take this into consideration. However, despite reporting similar barriers, motivators and support providers, only 48% of women with PCOS were active compared to 69% of women without the condition (χ^2^ = 0.050). Whilst standard scales and questionnaires can be used to measure barriers, motivators and support providers, there are clearly additional factors, unique to women with PCOS that are not being detected. In future research, investigation of barriers and motivators specifically related to PCOS would add value as it has been shown that hirsutism and acne can result in a negative self-image [25,51] which is a significant barrier to physical activity [52]. Women with PCOS have also been found to sweat more than other women during physical activity [53]. The physical symptoms, in addition to an increased prevalence of obesity in this population have been found to be associated with an increased level of self-consciousness during physical activity [51]. The development of a tailored psychometrically validated questionnaire to examine the specific barriers and motivators of physical activity for women with PCOS is needed. Considering the geographic location, cultural environment and other unique population factors may also be important when administering questionnaires of this nature.

Limitations of this research include that it relies on self-report, is cross-sectional in design, has uneven group numbers and has a limited measures of physical activity. Although both groups were recruited from the general community, more women with PCOS volunteered than women without PCOS and there were some demographic differences between groups with potential of volunteer bias leading to an under or over–estimation of effect. Also barriers and motivators to physical activity are assessed as frequencies rather than ranking and use a non-validated questionnaire with no available validated questionnaires in PCOS. Splitting the women into three age groups when assessing barriers, motivators and support providers does inherently reduce the variance associated with the measure. However, given the exploratory and hypothesis generating nature of the study, assessing barriers, motivators and support providers according to age assists in the planning of future research addressing the factors which help and/or hinder women with PCOS’ physical activity plans. Strengths of the study include a large, multiethnic community-based sample, incorporating both anxiety and depression, and including a control group, which provides formative data on physical activity as a component of first line management in PCOS.

## Conclusions

This study found that less women with PCOS were physically active compared to women without PCOS and that physical activity was associated with milder depression. While physical activity barriers, motivators and supports for women with PCOS are similar to those without PCOS, additional barriers in PCOS may limit engagement and sustainability of physical activity over the long term. This formative research will assist understanding of the interaction between mental health and active lifestyles for women with PCOS. Barriers, motivators and the need for support should be considered when prescribing and promoting of physical activity as a management strategy for PCOS. Future research may focus on eliciting specific physical activity barriers, motivators and support providers from women with PCOS in order to understand how to better prescribe physical activity as a management strategy to this population. Including theoretical frameworks in order to structure research may be advantageous, the development and use of validated questionnaires and of objective measures of physical activity may also strengthen findings in future research. Ultimately, current results support the need for more investigation regarding the physical and psychological impact of physical activity in PCOS to identify evidence-based strategies to optimise motivation for and engagement in sustainable physical activity.

## Abbreviations

PCOS: Polycystic ovary syndrome; BMI: Body mass index; TTM: Transtheoretical model; HADS: Hospital anxiety and depression scale; MANCOVA: Multivariate analysis of variance.

## Competing interests

This project is supported by a BRIDGES Grant from the International Diabetes Federation. BRIDGES, an International Diabetes Federation project, is supported by an educational grant from Lilly Diabetes.

## Authors’ contributions

MGH & HT were involved in the design of the study, acquisition of data and interpretation of results. NS was involved with design of the study and interpretation of results. LB and RP were involved in the analysis and interpretation. LB drafted the article and all authors contributed to the revisions and final version of the article. All authors have approved the final version of the article.

## Pre-publication history

The pre-publication history for this paper can be accessed here:

http://www.biomedcentral.com/1472-6874/14/51/prepub
